# Supplementary material for: Does Frenotomy Modify Upper Airway Collapse in OSA Adult Patients? Case Report and Systematic Review
Source: J Clin Med. 2022 Dec 27;12(1):201. doi: 10.3390/jcm12010201 (PMC9821269; doi:10.3390/jcm12010201)
Supplement: Supplementary file 1 [file jcm-12-00201-s001.zip › jcm-2092523-Supplementary/File S6-Prospero CRD42022360552 Protocol.pdf]

National Institute for Health Research

UNIVERSITY of York
Centre for Reviews and Dissemination

Systematic review

A list of fields that can be edited in an update can be found here

1. * Review title.

Give the title of the review in English

Lingual frenulum surgery in Adult patients with obstructive sleep apnea: a Systematic review protocol

2. Original language title.

For reviews in languages other than English, give the title in the original language. This will be displayed with the English language title.

English

3. * Anticipated or actual start date.

Give the date the systematic review started or is expected to start.

16/09/2022

4. * Anticipated completion date.

Give the date by which the review is expected to be completed.

15/10/2022

5. * Stage of review at time of this submission.

This field uses answers to initial screening questions. It cannot be edited until after registration.

Tick the boxes to show which review tasks have been started and which have been completed.

Update this field each time any amendments are made to a published record.

The review has not yet started: Yes

Review stage	Started	Completed
Preliminary searches	No	No
Piloting of the study selection process	No	No
Formal screening of search results against eligibility criteria	No	No
Data extraction	No	No
Risk of bias (quality) assessment	No	No
Data analysis	No	No

Provide any other relevant information about the stage of the review here.

6. * Named contact.

The named contact is the guarantor for the accuracy of the information in the register record. This may be any member of the review team.

Eduardo Javier Correa

Email salutation (e.g. "Dr Smith" or "Joanne") for correspondence:

Mr Correa

7. * Named contact email.

Give the electronic email address of the named contact.

ecorreaorl@gmail.com

8. Named contact address

Give the full institutional/organisational postal address for the named contact.

Bardenas 4 B Leganes - Madrid- 28915

9. Named contact phone number.

Give the telephone number for the named contact, including international dialling code.

+34665729403

10. * Organisational affiliation of the review.

Full title of the organisational affiliations for this review and website address if available. This field may be completed as 'None' if the review is not affiliated to any organisation.

Department of Otorhinolaryngology, Hospital Quiron Salud Marbella & Hospital Quiron Salud Campo de Gibraltar, Postal address, Avenida Severo Ochoa 22, Marbella, 29603 Malaga, Spain

Organisation web address:

https://www.quironsalud.es/marbella

11. * Review team members and their organisational affiliations.

Give the personal details and the organisational affiliations of each member of the review team. Affiliation refers to groups or organisations to which review team members belong. **NOTE: email and country now MUST be entered for each person, unless you are amending a published record.**

Dr Carlos O´Connor Reina. Co-Chair of Department of Otorhinolaryngology, Hospital Quiron Salud Marbella & Hospital Quiron Salud Campo de Gibraltar, Postal address, Avenida Severo Ochoa 22, Marbella, 29603 Malaga, Spain
Mr Eduardo Javier Correa. Department of Otorhinolaryngology, Hospital Quiron Salud Marbella & Hospital Quiron Salud Campo de Gibraltar, Postal address, Avenida Severo Ochoa 22, Marbella, 29603 Malaga, Spain
Dr Laura Rodriguez Alcalá. Department of Otorhinolaryngology, Hospital Quiron Salud Marbella & Hospital Quiron Salud Campo de Gibraltar, Postal address, Avenida Severo Ochoa 22, Marbella, 29603 Malaga, Spain
Dr Guillermo Plaza Mayor. Chief of Department of Otorhinolaryngology. Hospital Sanitas La Zarzuela & Hospital Universitario Fuenlabrada, Universidad Rey Juan Carlos, Madrid, Spain
Dr Peter Baptista Jardin. Otorhinolaryngology Department, Clinica Universitaria de Navarra, Pamplona, Spain
Diego Marcelo Conti. Otorhinolaryngology Department, Gent University Hospital, Gent, Belgium
Maria Teresa Garcia Iriarte. Otorhinolaryngology Department, Hospital Virgen de Valme, 41014 Sevilla, Spain
Antonio Moffa. School of Medicine, Campus Bio-Medico University, Unit of Integrated Therapies in Otolaryngology, Fondazione Policlinico Universitario Campus Bio-Medico, Rome, Italy

12. * Funding sources/sponsors.

Details of the individuals, organizations, groups, companies or other legal entities who have funded or sponsored the review.

No funding

Grant number(s)

State the funder, grant or award number and the date of award

No grant

13. * Conflicts of interest.

List actual or perceived conflicts of interest (financial or academic).

None

14. Collaborators.

Give the name and affiliation of any individuals or organisations who are working on the review but who are not listed as review team members. **NOTE: email and country must be completed for each person, unless you are amending a published record.**

15. * Review question.

State the review question(s) clearly and precisely. It may be appropriate to break very broad questions down into a series of related more specific questions. Questions may be framed or refined using PI(E)COS or similar where relevant.

In adult patients with ankyloglossia and obstructive sleep apnea, does lingual frenulum surgery impact on upper airway and OSA?

16. * Searches.

State the sources that will be searched (e.g. Medline). Give the search dates, and any restrictions (e.g. language or publication date). Do NOT enter the full search strategy (it may be provided as a link or attachment below.)

PubMed, Cochrane, Scopus, ProQuest.

Google Scholar (restriction: only thesis)

Web of Science (restriction: only scientific articles, and doctoral thesis)

17. URL to search strategy.

Upload a file with your search strategy, or an example of a search strategy for a specific database, (including the keywords) in pdf or word format. In doing so you are consenting to the file being made publicly accessible. Or provide a URL or link to the strategy. Do NOT provide links to your search **results**.

https://www.crd.york.ac.uk/PROSPEROFILES/360552_STRATEGY_20220915.pdf

Alternatively, upload your search strategy to CRD in pdf format. Please note that by doing so you are consenting to the file being made publicly accessible.

Yes I give permission for this file to be made publicly available

18. * Condition or domain being studied.

Give a short description of the disease, condition or healthcare domain being studied in your systematic review.

Ankyloglossia restricts tongue mobility, with consequent impairment in breastfeeding, deglutition, and speaking, and can interfere with craniofacial development and respiratory functions. The benefits of Ankyloglossia surgery in Adult patients with Obstructive Sleep Apnea (OSA) have not been studied yet. We questioned if frenulum surgery would modify the upper airway in these patients and improve their Sleep-disordered breathing (SDB)

19. * Participants/population.

Specify the participants or populations being studied in the review. The preferred format includes details of both inclusion and exclusion criteria.

Inclusion criteria:

- Adult patients (18 years old or more)

- Diagnosis of Obstructive Sleep Apnea by standardized sleep test and scoring.

- Clinical diagnosis of Ankyloglossia following a published and reproducible assessment tool / protocol

- Lingual frenulum surgery (no distinction of the surgical technique)

Exclusion criteria:

- Diagnosis of Obstructive Sleep Apnea not confirmed by standardized sleep test (e.g., questionnaires)

- Syndromic patients with craniofacial malformations

- Neurological comorbidities with swallowing or speech impairment

- Other/s upper airway surgical procedure/s performed concurrently

20. * Intervention(s), exposure(s).

Give full and clear descriptions or definitions of the interventions or the exposures to be reviewed. The preferred format includes details of both inclusion and exclusion criteria.

Lingual frenulum surgery for Ankyloglossia (with no distinction of the name given, e.g., frenectomy, frenulectomy, tongue-tie release)

21. * Comparator(s)/control.

Where relevant, give details of the alternatives against which the intervention/exposure will be compared (e.g. another intervention or a non-exposed control group). The preferred format includes details of both inclusion and exclusion criteria.

Pre-intervention versus post-intervention, or intervention versus non-exposed control group

22. * Types of study to be included.

Give details of the study designs (e.g. RCT) that are eligible for inclusion in the review. The preferred format includes both inclusion and exclusion criteria. If there are no restrictions on the types of study, this should be stated.

RCT, case report, case series, observational and clinical research studies

23. Context.

Give summary details of the setting or other relevant characteristics, which help define the inclusion or exclusion criteria.

Ankyloglossia diagnosed by protocolized and reproducible protocol

Obstructive sleep apnea diagnosed by a standardized sleep test.

24. * Main outcome(s).

Give the pre-specified main (most important) outcomes of the review, including details of how the outcome is defined and measured and when these measurement are made, if these are part of the review inclusion criteria.

The primary outcome intends to organize the literature regarding ankyloglossia surgery and sleep apnea by collecting case-report, case series, and observational and clinical research studies.

Measures of effect

Please specify the effect measure(s) for you main outcome(s) e.g. relative risks, odds ratios, risk difference, and/or 'number needed to treat.

25. * Additional outcome(s).

List the pre-specified additional outcomes of the review, with a similar level of detail to that required for main outcomes. Where there are no additional outcomes please state ‘None’ or ‘Not applicable’ as appropriate to the review

The secondary outcome looks at the impact of frenulum surgery in adult patients with obstructive sleep apnea and ankyloglossia by identifying intervention outcome measures, such as clinical protocolized upper airway evaluation, Quality of Life questionnaires, sleep studies, complementary image diagnosis or other standardized and reproducible evaluation tools.

Measures of effect

Please specify the effect measure(s) for you additional outcome(s) e.g. relative risks, odds ratios, risk difference, and/or 'number needed to treat.

26. * Data extraction (selection and coding).

Describe how studies will be selected for inclusion. State what data will be extracted or obtained. State how this will be done and recorded.

Mendeley Reference Manager for search references, SR-Accelerator to remove duplicates, and Rayyan for titles and abstracts reading.

Two researchers will independently screen the search results and title and abstract level blindly. In case of conflict, a third researcher will solve it.

Data to be extracted for eligible studies: study name, first author and year of publication, study design, number of patients included in the study, ankyloglossia assessment protocol, and outcomes.

27. * Risk of bias (quality) assessment.

State which characteristics of the studies will be assessed and/or any formal risk of bias/quality assessment tools that will be used.

We will use the ROBINS-I template from Cochrane to assess on the bias of the studies, involving:

- Confounding

- Selection bias

- Information bias

- Reporting bias

28. * Strategy for data synthesis.

Describe the methods you plan to use to synthesise data. This **must not be generic text** but should be **specific to your review** and describe how the proposed approach will be applied to your data. If meta-analysis is planned, describe the models to be used, methods to explore statistical heterogeneity, and software package to be used.

We will analyze the literature qualitatively and provide with a narrative synthesis of the findings from the included studies according to the type of population. We will also provide summaries of the intervention effects for each study. No meta-analysis will be carried out.

29. * Analysis of subgroups or subsets.

State any planned investigation of ‘subgroups’. Be clear and specific about which type of study or participant will be included in each group or covariate investigated. State the planned analytic approach.

No subgroups planned

30. * Type and method of review.

Select the type of review, review method and health area from the lists below.

Type of review

Cost effectiveness

No

Diagnostic
No

Epidemiologic
No

Individual patient data (IPD) meta-analysis
No

Intervention
No

Living systematic review
No

Meta-analysis
No

Methodology
No

Narrative synthesis
No

Network meta-analysis
No

Pre-clinical
No

Prevention
No

Prognostic
No

Prospective meta-analysis (PMA)
No

Review of reviews
No

Service delivery
No

Synthesis of qualitative studies
No

Systematic review
Yes

Other
No

Health area of the review

Alcohol/substance misuse/abuse
No

Blood and immune system
No

Cancer
No

Cardiovascular
No

Care of the elderly
No

Child health
No

Complementary therapies
No

COVID-19
No

Crime and justice
No

Dental
No

Digestive system
No

Ear, nose and throat
Yes

Education
No

Endocrine and metabolic disorders
No

Eye disorders
No

General interest
No

Genetics
No

Health inequalities/health equity
No

Infections and infestations
No

International development
No

Mental health and behavioural conditions
No

Musculoskeletal
No

Neurological
No

Nursing

No

Obstetrics and gynaecology
No

Oral health
No

Palliative care
No

Perioperative care
No

Physiotherapy
No

Pregnancy and childbirth
No

Public health (including social determinants of health)
No

Rehabilitation
No

Respiratory disorders
No

Service delivery
No

Skin disorders
No

Social care
No

Surgery
Yes

Tropical Medicine
No

Urological
No

Wounds, injuries and accidents
No

Violence and abuse
No

31. Language.

Select each language individually to add it to the list below, use the bin icon to remove any added in error.
English

There is not an English language summary

32. * Country.

Select the country in which the review is being carried out. For multi-national collaborations select all the countries involved.

Spain

33. Other registration details.

Name any other organisation where the systematic review title or protocol is registered (e.g. Campbell, or The Joanna Briggs Institute) together with any unique identification number assigned by them. If extracted data will be stored and made available through a repository such as the Systematic Review Data Repository (SRDR), details and a link should be included here. If none, leave blank.

34. Reference and/or URL for published protocol.

If the protocol for this review is published provide details (authors, title and journal details, preferably in Vancouver format)

Add web link to the published protocol.

Or, upload your published protocol here in pdf format. Note that the upload will be publicly accessible.

Yes I give permission for this file to be made publicly available

Please note that the information required in the PROSPERO registration form must be completed in full even if access to a protocol is given.

35. Dissemination plans.

Do you intend to publish the review on completion?

Yes

Give brief details of plans for communicating review findings.?

Review will be sent to a peer-reviewed indexed journal

36. Keywords.

Give words or phrases that best describe the review. Separate keywords with a semicolon or new line. Keywords help PROSPERO users find your review (keywords do not appear in the public record but are included in searches). Be as specific and precise as possible. Avoid acronyms and abbreviations unless these are in wide use.

ankyloglossia; tongue-tie; frenectomy; lingual frenulum; sleep apnea; snoring; obstructive sleep apnea;

apnea

37. Details of any existing review of the same topic by the same authors.

If you are registering an update of an existing review give details of the earlier versions and include a full bibliographic reference, if available.

38. * Current review status.

Update review status when the review is completed and when it is published.New registrations must be ongoing so this field is not editable for initial submission.
Please provide anticipated publication date

Review_Ongoing

39. Any additional information.

Provide any other information relevant to the registration of this review.

40. Details of final report/publication(s) or preprints if available.

Leave empty until publication details are available OR you have a link to a preprint (NOTE: this field is not editable for initial submission). List authors, title and journal details preferably in Vancouver format.

Give the link to the published review or preprint.
